# Feeding Blueberry Diets in Early Life Prevent Senescence of Osteoblasts and Bone Loss in Ovariectomized Adult Female Rats

**DOI:** 10.1371/journal.pone.0024486

**Published:** 2011-09-02

**Authors:** Jian Zhang, Oxana P. Lazarenko, Michael L. Blackburn, Kartik Shankar, Thomas M. Badger, Martin J. J. Ronis, Jin-Ran Chen

**Affiliations:** 1 Department of Pediatrics, University of Arkansas for Medical Sciences, Little Rock, Arkansas, United States of America; 2 Arkansas Children's Nutrition Center, Little Rock, Arkansas, United States of America; 3 Department of Physiology and Biophysics, University of Arkansas for Medical Sciences, Little Rock, Arkansas, United States of America; 4 Department of Pharmacology and Toxicology, University of Arkansas for Medical Sciences, Little Rock, Arkansas, United States of America; Pennington Biomedical Research Center, United States of America

## Abstract

**Background:**

Appropriate nutrition during early development is essential for maximal bone mass accretion; however, linkage between early nutrition, childhood bone mass, peak bone mass in adulthood, and prevention of bone loss later in life has not been studied.

**Methodology and Principal Findings:**

In this report, we show that feeding a high quality diet supplemented with blueberries (BB) to pre-pubertal rats throughout development or only between postnatal day 20 (PND20) and PND34 prevented ovariectomy (OVX)-induced bone loss in adult life. This protective effect of BB is due to suppression of osteoblastic cell senescence associated with acute loss of myosin expression after OVX. Early exposure of pre-osteoblasts to serum from BB-fed rats was found to consistently increase myosin expression. This led to maintenance osteoblastic cell development and differentiation and delay of cellular entrance into senescence through regulation of the Runx2 gene. High bone turnover after OVX results in insufficient collagenous matrix support for new osteoblasts and their precursors to express myosin and other cytoskeletal elements required for osteoblast activity and differentiation.

**Conclusions/Significance:**

These results indicate: 1) a significant prevention of OVX-induced bone loss from adult rats can occur with only 14 days consumption of a BB-containing diet immediately prior to puberty; and 2) the molecular mechanisms underlying these effects involves increased myosin production which stimulates osteoblast differentiation and reduces mesenchymal stromal cell senescence.

## Introduction

The development of degenerative bone disorders, such as osteoporosis, is associated with genetics and gene-environment interactions. Such disorders begin early in life but the complete phenotypic presentation can take decades to be manifested [1]. Hormone replacement therapy and medications have been the treatment of choice for conditions characterized by bone loss (such as postmenopausal osteoporosis) [2]. However, the extent to which these treatments can solve long-term problems of bone loss and resultant fragility is questionable. Attaining greater peak bone mass during critical bone-forming years could potentially delay the decline of bone mass to such an extent as to prevent or greatly delay the onset of osteoporosis [3], thus making the need for treatment either less likely or shorter, and significantly reducing health care costs. The majority of bone mass acquisition occurs between the ages of 12 and 18 years and results from substantial osteoblastic bone formation under control of an array of genes which interact with endogenous hormones and environmental factors [4].

In the search for alternative management of osteoporosis, potential preventive strategies through lifestyle changes involving diet and physical activity have recently received increased attention. For example, population-based studies indicate that fruit and vegetable intake are independent predictors of bone size in early pubertal children [5], [6]. Blueberries have recently been shown to promote osteoblastic bone formation without affecting normal growth in rapidly growing rodents, and this bone-promoting effect appears to be due to stimulation of osteoblastic differentiation caused by phenolic acid metabolites derived from BB polyphenols [7].

In general, bone formation is dependent on the activity and differentiation of osteoblasts; whereas, resorption of preexisting mineralized bone matrix by osteoclasts is necessary for bone remodeling [8]. In young rapidly growing animals, bone formation usually exceeds bone resorption resulting in bone accrual. Bone marrow stromal cells and periosteal osteoblast precursors are both potential sources of new osteoblasts [9]. Contact with bone collagenous matrix provides an important regulatory factor for these cells to function and differentiate [10]. Indeed, differentiation of mesenchymal stem cells toward tissue specific lineages is extremely sensitive to matrix qualities, such as elasticity [11]. It is still unclear whether accelerated degradation of collagens in bone matrix resulting from sex steroid deficiency results in secondary effects on osteoblast differentiation. In addition, cytoskeletal organization of mesenchymal stromal cells [12] or osteoblast precursors is thought to be critical for their differentiation into mature, functioning osteoblasts. To date, the relationship between sex steroid deficiency, cytoskeletal organization, bone and osteoblastic cell senescence is not well defined. Based upon our previous studies, we hypothesized dietary factors might affect bone matrix properties and organization of bone cell cytoskeleton, and thus prevent bone loss due to accumulation of senescent and dysfunctional osteoblasts or osteoblastic precursors which occurs under conditions of sex steroid deficiency.

Here we report that ovariectomy (OVX) triggers osteoblastic cell senescence in bone tissue along with decreased expression of myofibril genes. We have found that a diet containing BB prevents OVX-induced osteoblastic cell senescence and bone loss in adult female rats. We have shown that this protective effect requires only a very short period of time (between postnatal days 20 and 33) to be effective well into adulthood. We have demonstrated that early exposure of osteoblastic cells or mesenchymal stromal cells to BB diet maintains long-term cytoskeletal stability through regulation of myosin and Runx2 genes. Furthermore, we have identified molecular events of mesenchymal stromal cell senescence and osteoblast differentiation associated with OVX.

## Results

### Pre-pubertal consumption of a BB-containing diet prevents adult bone loss

**Figure 1:**
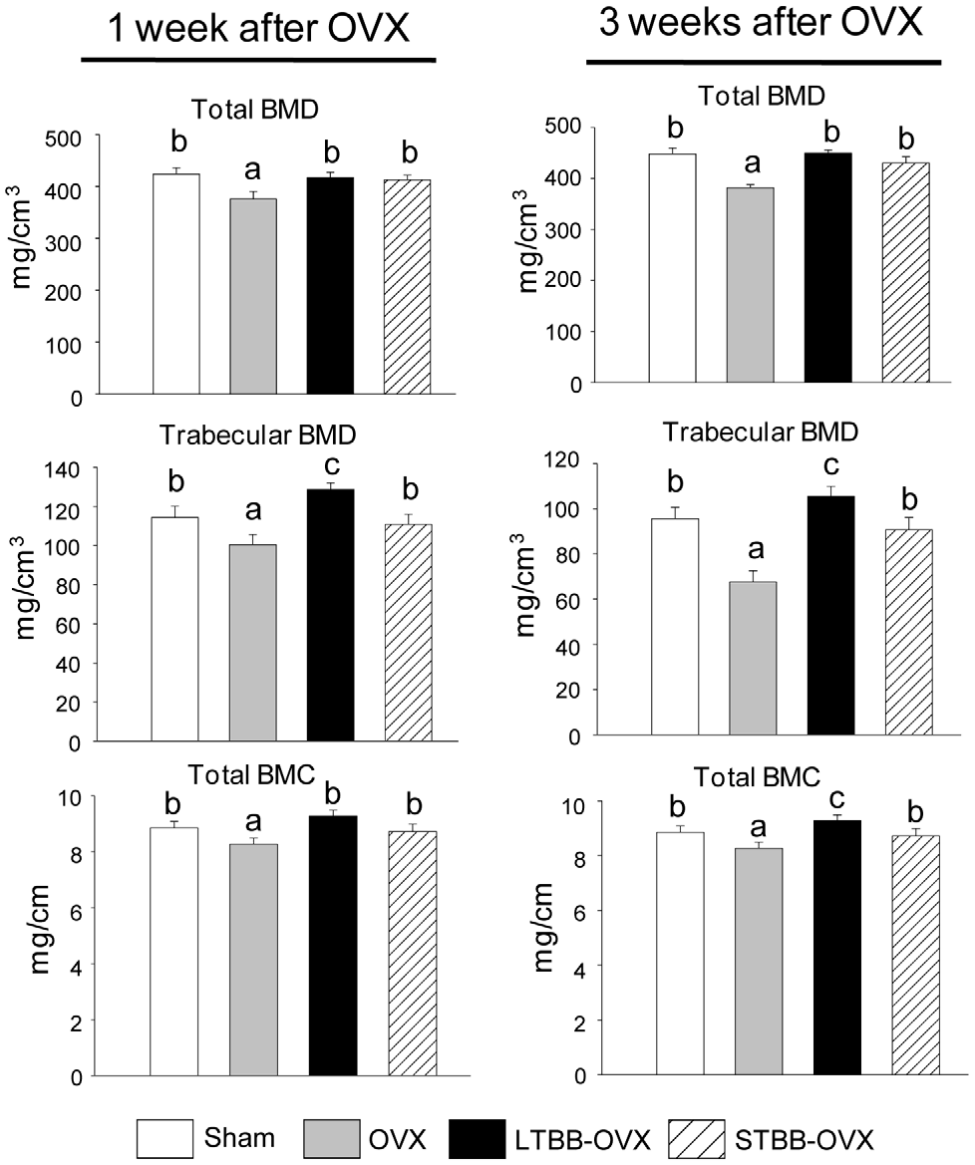
Continuous and early life short term blueberry diet prevents bone loss in adults. Parameters from pQCT analysis. Five consecutive slices from each rat tibia were scanned in a blinded manner. Data were analyzed from slices 3 and 4 (average). OVX, Ovariectomy; BMD, Bone mineral density; LTBB OVX, long term blueberry supplemented diet throughout experiment and ovariectomy. STBB OVX, short term blueberry diet for 14 days from weaning postnatal date 20 to PND 34, then switch to control diet and ovariectomy. Data are expressed as mean ± SEM (n = 8 per group). Significant differences indicated by p<0.05, a<b<c.

**Figure 2:**
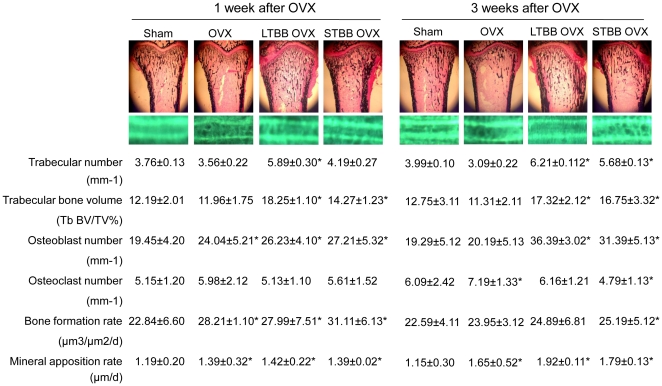
Static and dynamic histomorphometric parameters after OVX in female rats. Proximal tibia sections were histologically stained with Von Kossa and pictures were represented one from each group, and under Von Kossa staining, pictures were showing bone calcein double labeling. * p<0.05, versus Sham as determined by t-test. OVX; ovariectomy. LTBB; long-term blueberry. STBB; Short term blueberry.

We have previously reported that feeding a diet supplemented with BB to rats throughout life significantly promoted osteoblastic bone formation in rapidly growing male and female rats [7]. In the current study, in addition to long term BB feeding, additional groups of rats were weaned to the BB-containing diet starting on postnatal day 20 (PND20) and ending on PND34. They were fed a non-BB-containing control diet thereafter. This is referred to as short-term BB group. Our intent was to determine if a brief period of BB exposure early in the life of these rats would prevent bone loss when they were adults. Their bone parameters were compared to groups fed the control or BB-containing diets from PND20 until sacrifice. On PND60, all rats were ovariectomized and sacrificed 1 or 3 weeks later (see Figure S1 for experimental design). There were no significant differences in weight gain between comparable groups (Figure S1). After one or three weeks, OVX rats had decreased bone mass, including reduction in bone mineral density (BMD), and bone mineral content (BMC) compared with sham-operated animals (p<0.05) (Figure 1). Short-term feeding the BB-containing diet from PND20 to PND34 significantly blocked OVX-induced bone loss; whereas, long-term BB feeding increased bone mass compared to sham controls (Figure 1). Histomorphometric analysis of long bone sections indicated that the growth plates were not significantly altered by diet. Compared to sham-operated controls, BB-fed rats had increased (p<0.05) osteoblastic indices: including, trabecular bone volume; trabecular number; osteoblast numbers; and bone formation without significant changes in osteoclast parameters (Figure 2). After 3 weeks OVX, adult rats fed the BB diet from PND21 to PND34 had higher (p<0.05) levels of trabecular bone volume, osteoblast number and bone formation rate, but lower (p<0.05) numbers of osteoclasts compared to sham-operated or OVX controls (Figure 2).

Consistent with the results on bone mass and histomorphometric data, we found that one week after OVX, the bone formation marker osteocalcin and the bone resporption marker RatLaps were increased (p<0.05) in control diet-fed OVX rats compared with their sham controls (Table 1). Surprisingly, alkaline phophatase (ALP), was lower (p<0.05) in OVX animals compared to their sham control (Table 1). Osteocalcin remained higher in BB-fed groups compared to either sham or OVX control groups three weeks following OVX; whereas, ALP in BB-fed groups was equal to sham, but higher than the OVX control. In contrast, RatLaps was lower in BB-fed groups compared to control OVX controls, but higher than the sham operated controls (Table 1). It should to be noted that some of the effects of BB diet on bone in OVX rats are similar to previously published effects of estrogen replacement. However, the serum estradiol concentrations did not differ among OVX groups (data not shown). Importantly, these results indicate persistent bone formation effects associated with consumption of a BB-containing diet for a short period in early development. These data are clearly distinct from the effects of either anabolic or anti-remodeling agents reported previously [13] and suggest that adult peak bone mass may be enhanced by dietary intervention early in life.

**Table 1:** Serum bone turnover markers after OVX in female rats.

1 week after OVX				
	Sham	OVX	LTBB OVX	STBB OVX
Osteocalcin (ng/ml)	397.5±58.8^a^	485.2±47.2^b^	450.2±19.8^b^	453.0±35.6^b^
ALP (µU/min)	1.16±0.13^c^	0.80±0.14^a^	1.07±0.14^b^	1.06±0.18^b^
RatLaps (ng/ml)	19.1±1.9^a^	27.4±2.2^c^	20.4±3.8^a,b^	23.1±0.8^b^

3 weeks after OVX				
	Sham	OVX	LTBB OVX	STBB OVX
Osteocalcin (ng/ml)	377.5±30.5^a^	377.1±47.2^a^	433.3±38.8^b^	425.0±20.8^b^
ALP (µU/min)	1.00±0.18^b^	0.81±0.17^a^	1.08±0.21^c^	1.01±0.20^b^
RatLaps (ng/ml)	15.5±1.1^a^	26.5±1.6^c^	21.1±1.6^b^	22.2±2.1^b^

P<0.05, a<b<c as determined by one way ANOVA followed by Student-Newman-Keuls post hoc analysis for multiple pairwise comparisons. OVX; ovariectomy. LTBB; long-term blueberry. STBB; Short term blueberry.

### Myosin in bone is a target for prevention of OVX-induced bone loss by BB

**Figure 3:**
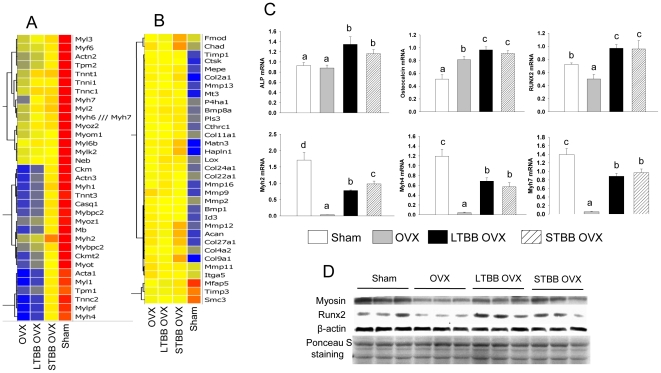
Myosin is the target gene for dietary BB effects on bone in OVX animals. Microarray profiling of bone cell transcripts in RNA from bone with or without BB and OVX. (A), Cluster of myosin related genes and, (B), cluster of collagen turnover related genes. Results are presented as fold changes relative to sham control. Color changes from red to yellow and blue represent decreases in gene expression. (C), Real-time PCR analysis for mRNA of ALP, osteocalcin, Runx2and myosin 2, 4, 7 in bone with different diets and 3 weeks following OVX in female rats. Data are expressed as mean ± SEM (n = 8 per group). Means with different letters differ significantly from each other at p<0.05, a<b<c. (D), Western analysis of myosin and Runx2 in bone from four different diet groups after 3 weeks of OVX, membrane ponceau S staining for protein loading control. Eight to nine samples in each group were randomly pooled into three samples per group are presented for microarray analyses.

**Figure 4:**
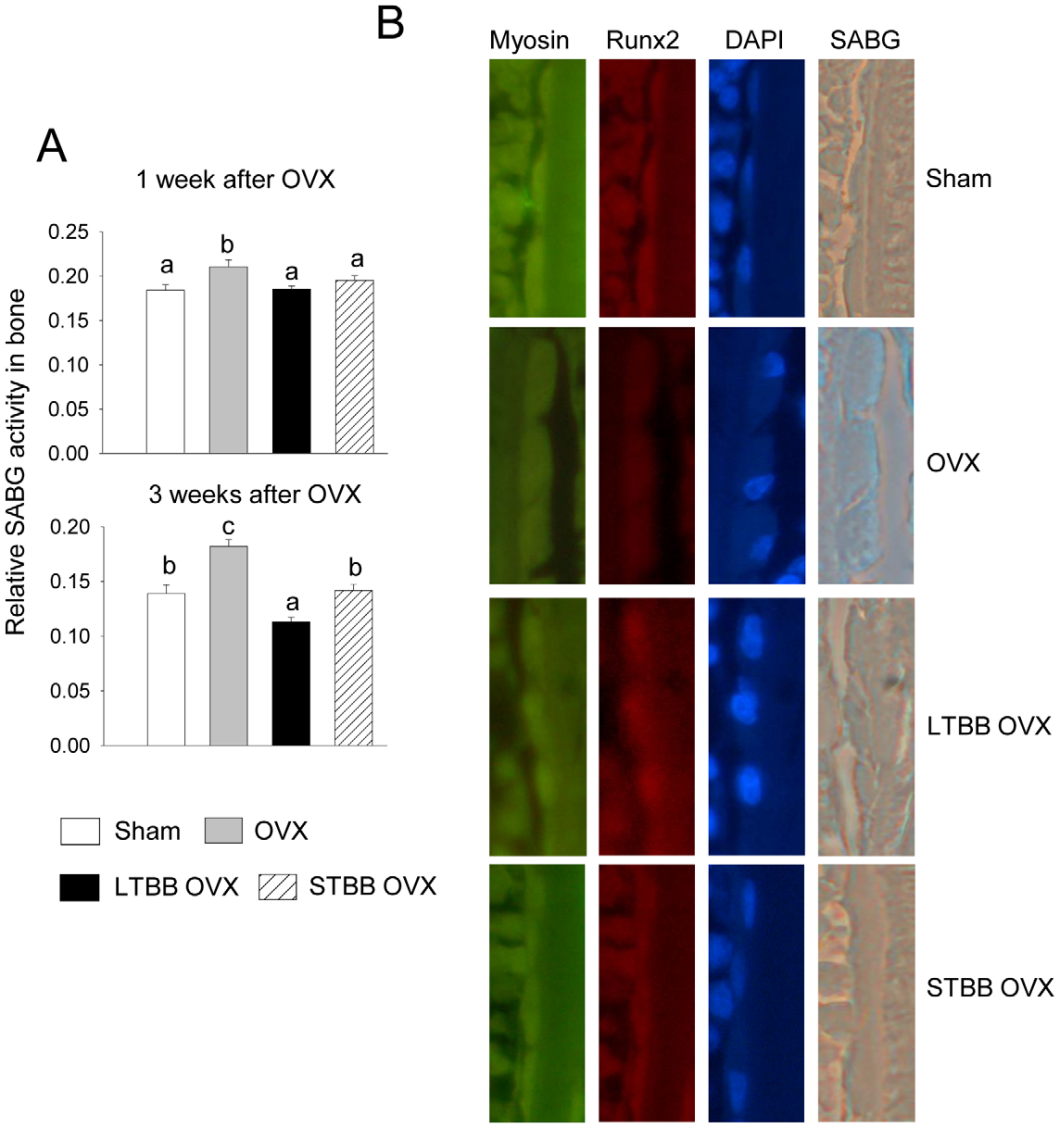
BB diet prevents OVX-induced acute bone loss and osteoblastic cell senescence. (A), Senescence associated beta-galactosidase (SABG) activity analyses in total proteins isolated from bone with four different diet groups. Data are expressed as mean ± SEM (n = 8 per group). Means with different letters differ significantly from each other at p<0.05, a<b<c. (B), Triple staining of immune-staining for myosin and Runx2 and SABG activity staining in long bone sections from four different diet groups 3 weeks after OVX. Pictures are showing typical bone surface osteoblasts from sagittal section under 20 x magnifications, Green stains for myosin, red stains for Runx2 and blue stains for SABG and DAPI staining for nucleus.

During early life, substantial bone formation is regulated by an array of genes which interact with endogenous hormones and environmental factors [14]. In an effort to determine which target genes are interacting with dietary factors in BB to protect against OVX-induced bone loss, mRNA from long bone of rats sacrificed 3 weeks post-OVX was studied using Affymetrix gene arrays. These analyses revealed a cluster of genes containing cell cytoskeletal motor proteins, particularly myosin, that was down-regulated in bone in the OVX control group compared with the sham-operated group (Figure 3A); whereas, another cluster of genes related to collagen turnover was up-regulated (Figure 3B). These data suggest a compensatory response to high bone turnover and bone formation after OVX. Feeding BB diets, particularly the short-term early in life, inhibited OVX-induced down-regulation of myosin-related genes (p<0.05), although BB did not completely block the OVX effect on myosin expression. For example, myosin 4 and 7 were down-regulated 400-fold in the control diet-fed OVX group compared with their sham-operated animals, and BB diet prevented more than 75% of this reduction. In contrast, the BB diet did not inhibit OVX-induced up-regulation of genes related to collagen synthesis, but rather enhanced expression of these genes above that seen OVX controls (Figure 3B). This suggests that BB promoted osteoblast differentiation or increased osteoblast activity. Real-time RT-PCR confirmed that gene expression of three subtypes of myosin, i.e. 2, 4 and 7, known as nonmuscular myosins, were profoundly reduced in the OVX controls. Furthermore, using real-time RT-PCR, we found that Runx2 expression was lower, osteocalcin expression was higher, and ALP expression was unchanged in samples from the OVX control group compared with their sham-operated control (p<0.05; Figure 3C). All three genes were increased (p<0.05) in OVX animals fed BB diets compared with sham-operated controls (Figure 3C). Myosin and Runx2 protein expression patterns were confirmed using Western blotting (Figure 3D). These results prompted us to explore the true significance of inhibition of OVX-induced down-regulation of myosin in bone by BB diet. Cell senescence-associated beta-galactosidase (SABG) activity, a biomarker and one of phenotypes of senescent tissues or cells [15] was examined from total protein isolated from bone. It was increased (p<0.05) one and three weeks following OVX compared with sham-operated controls (Figure 4A). SABG activity in BB-fed rats one week post-OVX approximated levels in sham-operated controls (Figure 4A). SABG activity three weeks after OVX in animals fed BB diet long-term was lower (p<0.05) than sham-operated controls, while rats fed the BB diet of short-term early in development did not differ from the sham-operated control group (Figure 4A). These results are reflected in the bone mass data presented in Figure 1.

We next examined whether there are associations between osteoblast senescence, myosin and Runx2 expression in bone three weeks after OVX with or without BB diet using triple staining of SABG activity staining, myosin and Runx2 immunostaining. We found that osteoblasts on the surface of bone from OVX animals showed relatively more staining of blue for SABG, less myosin and Runx2 staining, and were flattened and increased in size compared with those from sham-operated animals (Figure 4B). Cells from OVX animals fed the BB diet either for a short-term early in life or over a long-term showed no obvious differences compared with sham-operated controls (Figure 4B) (Figure S2).

### Serum from BB diet-fed rats induced osteoblastic cell commitment and differentiation involving myosin

**Figure 5:**
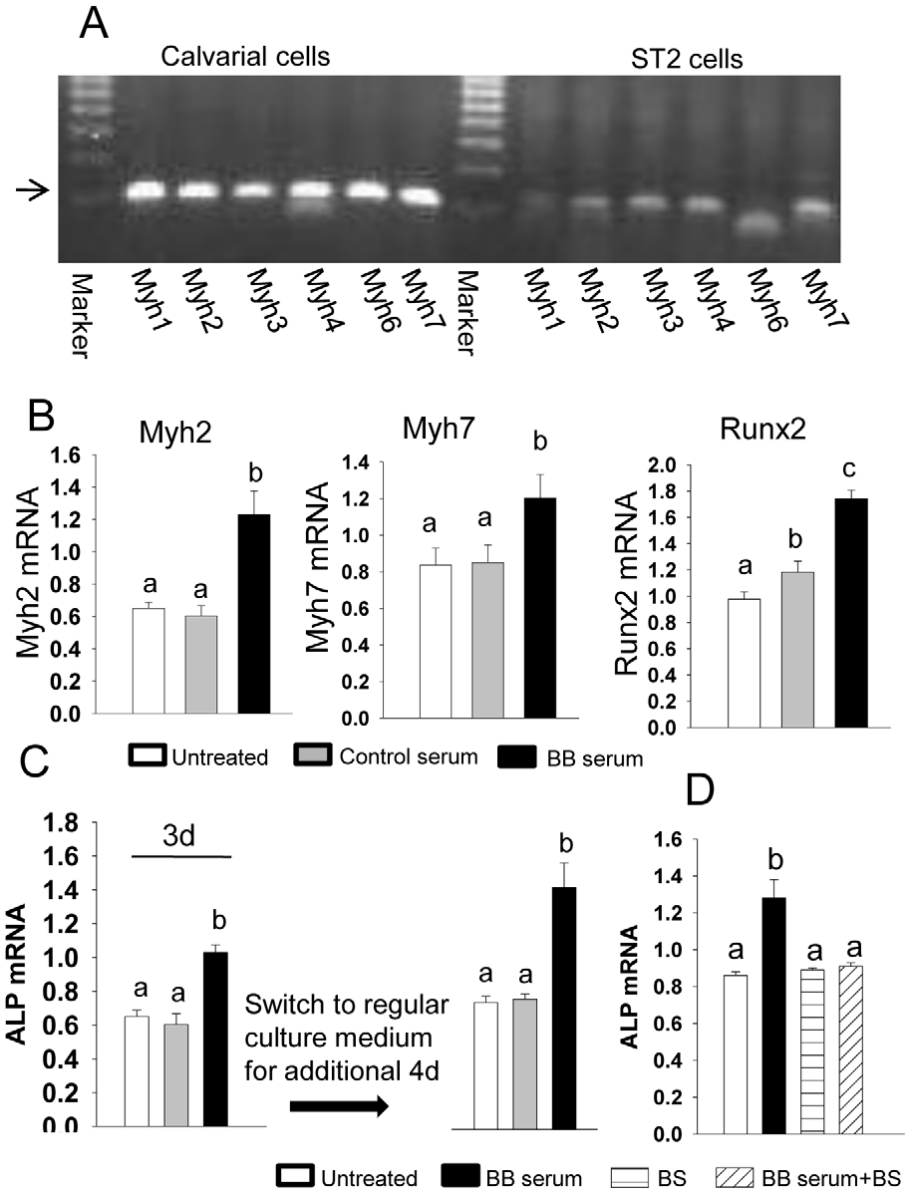
Serum from BB diet intact animals stimulates osteoblast commitment and differentiation. (A), Regular RT-PCR analysis for expressions of myosin1, 2, 3, 4, 6 and 7 in both calvarial osteogenic cells and ST2 cells. (B), Real-time PCR analysis for mRNA expression in mouse origin bone marrow stromal ST2 cells after cells were treated with 2% BB diet animal serum for 3d. (C), Real-time PCR analysis for ALP mRNA expression in mouse origin bone marrow stromal ST2 cells after cells treated with 2% BB diet animal serum for 3d, and then switched to regular cell culture medium for an additional 4d. (D), Real-time PCR analysis for ALP mRNA expression in mouse origin bone marrow stromal ST2 cells after cells treated with 2% BB diet animal serum, blebistatin 20 µm and their combination for 3d. Data are expressed as mean ± SEM (triplicates). Means with different letters differ significantly from each other at p<0.05, a<b<c.

We believe tissue-specific dietary effects are mediated by bioactive compounds derived from the BB diet that appear in the peripheral circulation following digestion. Serum from rats fed the BB diet (BB serum) was used to treat osteogenic and osteoblast progenitor cells *in vitro* to test our hypothesis. To obtain pretreatment measures, we first determined myosin expression in osteogenic rat fetal calvarial cells and mouse-derived bone marrow mesenchymal stromal ST2 cells. Based on previous microarray analysis (presented in Figure 3), myosin 1–4, 6 and 7 were profoundly down-regulated in bone from OVX controls. We then checked these 6 subtypes of myosin expression in pure osteoblastic cells using regular PCR and found they were all highly expressed in calvarial cells relative to their expression in ST2 cells (Figure 5A). ST2 cells (and data from calvarial cells were presented in Figure S3) were then treated with 2% rat serum from either control or 14 d BB diet-fed female rats sacrificed on PND34. Treatment of ST2 cells with BB serum for 72 h increased (p<0.05) gene expression of the transcription factor Runx2 and increased (p<0.05) all myosin subtype gene expressions (Figure 5B). Consistent with *in vivo* bone mass data, we found that after treatment of ST2 cells with BB serum for three days and return to regular cell culture medium for additional 4 days, ALP gene expression remained significantly higher than those in cells treated with serum from control diet-fed rats (Figure 5C). These data indicate that BB serum is able to induce mesenchymal stromal cell commitment to osteoblastic cell lineage. Using Affymetrix gene array analyses, transcriptional profiles of osteogenic markers, from early commitment markers through mid/late development markers, were consistent with results from real-time PCR analysis (Figure S4). Surprisingly, in the presence of blebbistatin, a non-muscle myosin 2 specific inhibitor, the osteogenic effects of BB serum was blocked (Figure 5D). These data suggest that myosin, or at least non-muscular myosin, is involved in BB diet-induced osteoblast commitment or differentiation.

### Osteoblastic cell shape maintenance and senescence prevention by dietary BB through myosin and Runx2

**Figure 6:**
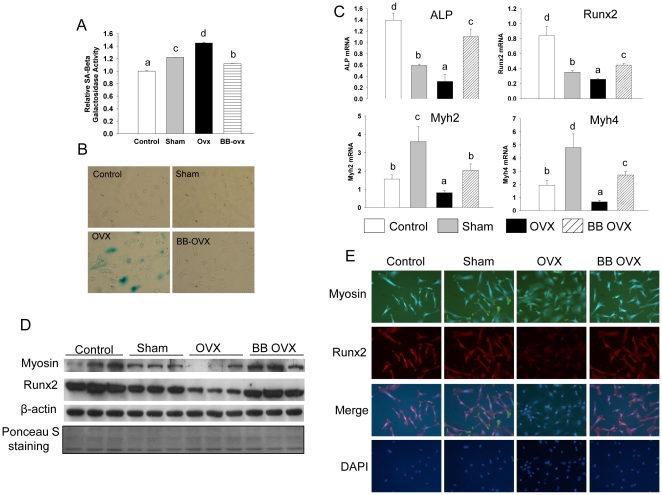
BB diet is able to maintain osteoblastic cell shape and prevent OVX-induced cell senescence. (A), After calvarial cells treated one time with 2% of PBS (control), serum from three weeks of sham operated, OVX and long term BB plus OVX (BB OVX) rats for 3d, cell cultures were switched to a regular medium until to last passage 9. Proteins were isolated from passage 9 cells, and senescence associated beta-galactosidase activity was measured. (B), Passage 9 cells were stained with senescence associated beta-galactosidase activity; cells with blue color are positively stained cells. (C), RNA were isolated from passage 9 cells, and mRNA expressions of ALP, Runx2 and myosin2, 4 were carried out by real-time PCR. (D), Western analysis of myosin and Runx2 expressions in passage 9 cells. (E), Myosin and Runx2 double immune-staining was performed on passage 9 cells, myosin stained green and Runx2 stained red (10x). Data are expressed as mean ± SEM (triplicates). Means with different letters differ significantly from each other at p<0.05, a<b<c<d.

**Figure 7:**
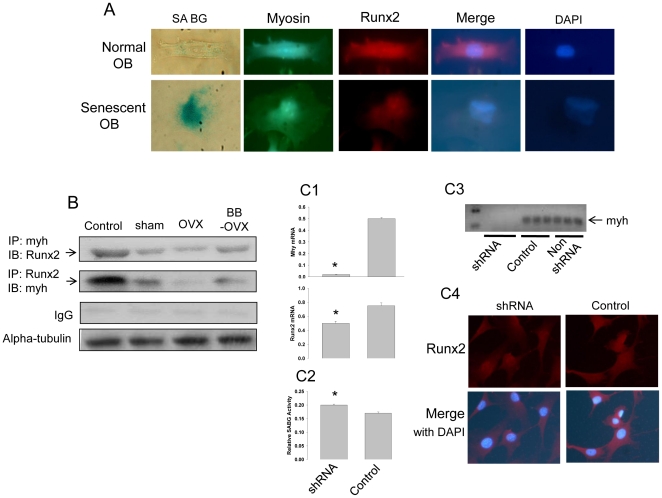
Myosin and Runx2 interaction determines osteoblastic cell senescence. (A), A single morphologically normal and senescence associated beta-galactosidase (SABG) negative osteoblastic cell was compared with a SABG positive cell from OVX rat serum treated on myosin and Runx2 expression and localization. Myosin stained green and Runx2 stained red with antibody immune staining. (B), Coimmunoprecipitation of myosin and Runx2 in lysates of calvarial cells cultured and treated with 2% of sham operated, OVX and continuous BB diet plus OVX (BB OVX) rat serum. (C1), Myosin and Runx2 mRNA expressions after silencing of the myosin gene using shRNA myosin in ST2 cells. Data are expressed as mean ± SEM (triplicates). *, p<0.05, significantly different than control non shRNA normal culture medium. (C2), SABG activity in ST2 cells with or without shRNA myosin. Data are expressed as mean ± SEM (triplicates). *, p<0.05, significantly different than control. (C3), Regular PCR to check myosin expression after shRNA in ST2 cells. (C4), Runx2 immuno-staining (stained red) after shRNA myosin in ST2 cells.

We have shown above that BB diet short-term in early life protected against OVX-induced bone loss in adults, and that this effect may be due to the ability of dietary BB to suppress OVX-induced osteoblast senescence associated with reduced myosin expression. To further confirm this and *in vivo* data, we utilized fetal calvarial cells (and ST2 cells as data presented in Figure S5). Cells were treated for 3 days with 2% rat serum taken from adult female sham-operated or OVX (3 weeks post-OVX) rats fed non-BB-containing diets. Similar groups of cells were treated with serum from adult female rats fed long-term the BB diet from weaning and sacrificed 3 weeks following OVX. After three days of serum treatment, all cells were then cultured in normal medium and cell cultures were continued in the presence of regular cell culture medium for nine passages (Figure S6A). We identified the total population of cells that were confluent every 5d to 6d. We found that cells cultured in control medium or exposed to serum from sham-operated or OVX controls for three days at the beginning of the experiment gradually and significantly descended in number, particularly after passage four (Figure S6B). In contrast, even after four passages, cells exposed to rat serum from BB-fed OVX rats, had numbers which remained relatively similar to their previous passage (Figure S6B). Although, after nine passages, fetal calvarial cells were still able to proliferate and did not reach crisis, cells treated with serum from control diet-fed OVX rats had higher (p<0.05) SABG activity (Figure 6A) and more SABG-stained, morphologically-altered cells (Figure 6B) compared with the other three treatments. mRNA and protein were isolated from cells after 9 passages following all 4 treatments. Gene expression of the osteoblast differentiation markers ALP and Runx2 were higher (p<0.05) in cells pretreated with serum from OVX BB diet-fed rats compared to those cells pretreated with serum from OVX control rats (Figure 6C). This was accompanied by higher (p<0.05) gene expression of myosin (Figure 6C). Moreover, we have observed consistent and significant differences of Runx2 and myosin gene encoded protein expression using Western blot analyses (Figure 6D). As in our previous *in vivo* analysis, myosin and Runx2 double immuno-staining was performed on passage nine cells that had been subjected different serum treatments for only three days at beginning of the experiment. We found that expression of Runx2 was lower (p<0.05) in cells from OVX control diet rat serum treatment compared with the other treatments (Figure 6E). Clearly, decreased expression of Runx2 in cells treated with serum from OVX control rats was accompanied by lower (p<0.05) expression of myosin (Figure 6E). Interestingly, SABG-stained positive cells treated with serum from OVX control rats were larger, flatter and rounder than SABG-negative cells from the OVX long term BB diet rat serum-treated group, where the cells were smaller and spindle-shaped (Figure 7A). The SABG positive cell not only showed less expression of myosin and Runx2 relative to the SABG negative cell, but the limited expression of Runx2 was specifically confined to the nucleus (Figure 7A). These observations suggested that myosin expression in osteogenic cell may play a role in shuttling Runx2 from cytoplasm to nucleus and vice versa. Myosin-dependent nuclear-cytoplasmic shuttling of Runx2 may be particularly important for providing a mutual link between myosin and signal transduction cascades that ultimately control transcription of downstream targets in osteoblastic cells. An association between myosin and Runx2 was observed using co-immuno-precipitation, and serum from OVX control rats decreased (p<0.05) this myosin and Runx2 complex (Figure 7B). Consistently, knockdown of the myosin gene in ST2 cells resulted in reduced expression of Runx2 mRNA, and less potential for cells to differentiate into osteoblasts even in the presence of BB serum (Figure 7C). Following treatment with shRNA to myosin, ST2 cells expressed greater (p<0.05) SABG activity (Figure 7C2), indicating increased senescence.

## Discussion

By treating animals with BB-supplemented diets either for a short term prior to puberty or long term continuously from weaning through adulthood, we have discovered that bone loss in adulthood resulting from sex-steroid deficiency (herein referred as OVX-induced bone loss) could be prevented. Our results suggest that the protective effect of dietary BB against bone loss is due to its ability to increase cytoskeletal organization of osteoblastic cells, which allows cells to maintain their appropriate differentiation potential and function and which makes them resistant to senescence.

We have presented results supporting a novel mechanistic paradigm of how OVX-induced bone loss proceeds and how dietary BB stimulates bone formation through enhancing myosin expression in osteoblasts and their precursors. Our findings indicate that organization of an appropriate cytoskeletal structure may drive stem cells to differentiate into a functional and tissue-specific mature cell. In particular, motor proteins in non-muscle cells, such as myosin described in this report, may act as a shuttle between cytoplasm and nucleus for essential osteoblastic cell differentiation factors such as Runx2 (the runt domain-containing transcription factor). The presence of this shuttle in osteoblastic cell precursors in early life is particularly important to preserve the fidelity of stromal cells for their differentiation potential later in development. We have demonstrated Runx2 is a critical mechanistic factor linking dietary BB, expression of cytoskeletal proteins and lineage-specific cell fate, proliferation and growth. Interestingly, our data suggest that OVX-induced degradation of bone matrix may secondarily effect osteoblastic cell differentiation and function through down-regulation of myosin expression leading to a second phase of bone loss after OVX. This extended effect has not previously been documented, and suggests a novel mechanistic explanation for the extent of OVX-induced bone loss which may involve metabolic programming, perhaps through epigenetic modulation.

Theoretically, the lower the peak bone mass acquired by the menopause, the higher the risk of osteoporosis later in life and the earlier it begins. Accordingly, with all other factors being approximately equal (such as, general health and age at menopause), women with significantly greater peak bone mass at menopause would be expected reach osteoporotic bone conditions later in life. The age at which bone is acquired and the diet are two critical factors thought to be important in determining peak bone mass. For example, it is known that bone formation during childhood and adolescence is very critical, and nearly half of the peak bone mass is acquired during these years [16], [17]. Aside from known dietary components required for bone development and health (such as, adequate protein, calories, calcium, phosphorus, and vitamin D), certain other dietary factors such as phytochemicals may increase bone development and bone mass which ultimately reduce the risk or degree of osteoporotic bone loss later in life. We have previously reported that weaning rats with only 2 weeks of BB supplemented diet had remarkable increases in bone density and bone mineral content without affecting normal growth and there were no gender differences [7]. It was concluded that dietary BB had a positive effect on building peak bone mass. The significance of such increased peak bone mass in early life by BB for buffering bone loss in an adult model of estrogen deficiency (ovariectomy) is the topic of this report.

Bone loss occurs with increasing age in females is directly linked to loss of ovarian function [18]. The pathophysiology of this “ovary-related” bone loss is very complicated and cannot be simply explained by either increased bone resorption or decreased bone formation [19]. Currently, treatment of postmenopausal osteoporosis has been with drugs and hormone therapy, but some of the approved treatments have specific negative side effects such as mastalgia and increased risks of breast cancer and endometrial hyperplasia. Furthermore, none of these treatments has been able to satisfactorily solve long-term problems of bone loss. The results presented in the current study suggest that early life nutrition may promote higher bone mass/quality which is maintained into adulthood and protects against sex-steroid deficiency-induced bone loss: a condition modeling postmenopausal bone loss in women. Consistent with our findings, previous clinical and animal studies have also demonstrated that increased bone mass in early life produced by either appropriate nutrition or exercise persist into early adulthood [20], [21], [22]. A recent rodent study showing that neonatal mice treated with soy isoflavones produced significant increases in adult bone quality [23]. Although the underlying mechanisms still need to be defined, the above cited studies are consistent with our findings.

Moreover, our findings indicate that bone marrow mesenchymal stromal cells can be programmed postnatally by a dietary factor to commit and differentiate into functional osteoblasts in later adult life. In adults, appropriate bone remodeling is a fundamental process in which new bone formation is tightly associated with bone resorption to support and preserve skeletal health. Ovarian dysfunction results in increases in bone resorption which trigger transient compensatory increases in bone formation to maintain bone remodeling at a set level. However, bone formation is eventually exceeded by increased bone resorption, a net bone loss results. The effects of dietary BB on osteoblastogenesis and osteoclastogenesis appear similar to that of estrogens. However, the serum estrogen levels did not differ between BB-fed rats and control rats. Therefore, it is unlikely that the effect of BB diet on bone in OVX animals is due to increased levels of estrogen or its related compounds.

One therapeutic approach has used agents with anabolic properties to increase bone formation such as PTH (parathyroid hormone) to treat ovary dysfunction-induced bone loss. Similar bioactive compounds in serum associated with consumption of a BB-containing diet are still under investigation in our laboratory. Dietary BB-induced serum phenolic acids [7] may play a key role in protecting against OVX-induced bone loss by stimulating bone formation. Nonetheless, the additional ability of BB diets to prevent bone and osteoblastic cells from acutely entering senescence after OVX is an interesting and novel finding. Previous studies have suggested that osteoblast apoptosis was increased in addition to increased osteoclast activity after OVX in female rodents [18]. It is not known whether osteoblast apoptosis is an important consequence of increased bone remodeling after OVX. We believe that the rapid entry into cell senescence may be an important fate of osteoblasts after OVX. As we have demonstrated in this report, the activity of senescent osteoblasts was significantly lower, and their cell shape may be changed, but they can still identified as osteoblasts. This is consistent with our histomorphometric observation which showed increased osteoblast numbers in OVX animals particularly after 1 week. BB diet may increase the life span of osteoblastic stromal stem cells and progenitor self renewal, and may also enhance osteoblast commitment and differentiation. Determining the mechanisms whereby blueberries prevent the OVX-induced osteoblast senescence requires further elucidation. Furthermore, the mechanisms by which OVX triggers osteoblasts to rapidly enter senescence may be fundamental for understanding the true pathophysiology of OVX-induced bone loss.

Microarray data analyses revealed a cluster of myofibril gene expression particularly myosin related genes that were remarkably down-regulated after OVX in total RNA isolated from bone. Dietary BB significantly inhibited OVX-induced suppression of myosin expression in bone. Furthermore the expression of myosin, particularly non-cardiovascular myosin, is broad and the spectra of their functions have not been fully described in non muscular tissue. We believe that myosin not only plays a role in supporting cell structure, but also conveys some molecular signals in cells from non muscular tissue, and bone forming osteoblasts or their precursors are unlikely to be an exception. We have shown that at least 6 myosin subtypes are highly expressed in osteogenic cells and osteoblastic stromal cells. Reduction of myosin expression in skeletal muscle after OVX has been reported [24], and interestingly, during osteoclastogenesis, myosin IIA may be temporarily suppressed [25]. Moreover, recent studies have suggested that myosin may play a role in driving a cell to remodel, and eventually influence stromal cells to differentiate into tissue-specific cells [26]. We have found evidence that myosin expression in osteogenic cells and mesenchymal stromal cells may be associated with maintaining Runx2 expression and shuttling this well known essential osteoblast differentiation transcription factor between cell cytoplasm and nucleus. Runx2 has been shown to establish and maintain cell identity and convey phenotypic information through successive cell division or exit in progeny cells [27], and it is required for osteoblast differentiation [28]. The phenomenon of Runx2 shuttling between the cytoplasm and the nucleus has been reported and may depend on microtubules in cancer cells [29]. Our data in osteogenic cells indicated an association of myosin with Runx2. Treatment of osteogenic cells with serum from OVX control diet rats decreased both myosin and Runx2 complex, but treatment with serum from BB-fed rats increased this complex. To maintain this complex at the appropriate functional level may be particularly important, because significantly reduced expression of Runx2 level will not only suppress osteoblast differentiation, but also result in a cell entering the senescent stage. This is consistent with previous published evidence showing that Runx2 interacts with histone deacetylase 6 to repress the p21 gene, known as a downstream effecter of p53 that is associated with cell senescence [30] Moreover, it has been shown that myosin VI DNA damage is p53-dependent [31]. Consuming BB early in life contributes to development or maintenance of the myosin-dependent Runx2 shuttle, and this may also be important to preserve the ability of mesenchymal stromal cells to differentiate into functional osteoblasts later in life under inappropriate stress. However, the mechanistic associations among specific subtypes of myosin, Runx2 and p53 in osteoblastic cells need to be further elucidated.

In conclusion, we have reported that ovariectomy triggers acute bone loss and osteoblastic cell senescence in association with decreased expression of myofibril genes. Consuming a BB diet for just a short period in prepubertal life or continuous consumption starting a weaning both prevented OVX-induced bone loss and osteoblastic cell senescence in adult female rats. We have demonstrated that early exposure of osteoblastic cells or mesenchymal stromal cells to dietary BB maintains long-term cytoskeletal stability by regulation of myosin and Runx2 genes. These results show a significant prevention of OVX-induced bone loss by early life dietary BB, and further provide insight into molecular events of mesenchymal stromal cell senescence and osteoblast differentiation associated with OVX.

## Materials and Methods

### Animals and Diets

Time-impregnated female Sprague-Dawley rats (n = 6) (Harlan Industries, Indianapolis, IN) gestational day 4 were individually housed in an Association for Assessment and Accreditation of Laboratory Animal Care-approved animal facility at the Arkansas Children's Hospital Research Institute with constant humidity and lights on from 06:00-18:00 hrs at 22°C. All animal procedures were approved by the Institutional Animal Care and Use Committee at University of Arkansas for Medical Sciences (UAMS). The approval ID for this study is 2473. Pregnant rats were fed AIN-93G diets [32]. Litters from these dams were culled to 5 male and 5 female pups. Pups at post-natal day 20 (PND20) were randomly assigned (10 per group) to AIN-93G diets with or without blueberry (BB) supplementation until PND34 as the short-term BB group, and BB diet from PND20 throughout life as the long-term BB group. Each group of animals and their detailed diets, surgery and age are presented in Supplemental Figure 1. Freeze-dried whole BB (Vaccinium angustifolium) powder (Hi-Actives Wild Blueberry) was kindly provided by VDF/FutureCeuticals, Momence, IL. AIN-93G supplemented with 10% freeze-dried BB powder (10%BB) was made by Harlan Teklad (Madison, WI). To eliminate caloric density as a confounding variable, all diets were formulated to be isocaloric and isonitrogenous. The diets contained the National Research Council nutrient recommendations and the same calcium and phosphorus levels [33]. Animals were weighed every other day.

### Bone peripheral quantitative computerized tomography (pQCT) and histomorphometry

At sacrifice the right rear tibia was removed and frozen in liquid nitrogen. pQCT scans were performed on individual tibial bones from each rat using a STRATEC XCT 960M unit (XCT Research SA, Norland Medical Systems, Fort Atkins, WI) with software version 5.4. The detail of pQCT scanning was described previously [34]. Several key points were briefly summarized below. The position for pQCT scanning was defined at a distance from the proximal tibia growth plate. All analyses were conducted in a blinded fashion. Five consecutive slices separated by 1 mm (1 through 5, 1 being most distal) were scanned for each tibia beginning immediately below the tibial growth plate. Data from slice 2 and 3 were combined and presented in Figure 1. A threshold of 470 mg/cm^3^ was used to distinguish cortical bone, and 107 mg/cm^3^ was used to distinguish cancellous bone through the experiment. Tibial bone mineral density (BMD) was separated into total and trabecular and cortical compartments and bone mineral content (BMC) were automatically calculated and color images were generated. At sacrifice, calcein-labeled, left rear tibial bones were removed and fixed, sequential dehydration was carried out using different concentrations of alcohol. Proximal tibial bone samples were embedded, cut and von Kossa, tetrachrome and Masson stained by Histology Special Procedures. For histomorphometric analysis, sections were read in a blinded fashion. Parameters of cancellous and cortical bones in the proximal tibia and tibial shaft were measured with a digitizing morphometry system, which consists of an epifluorescent microscope (model BH-2, Olympus), a color video camera, and a digitizing pad (Numonics 2206) coupled to a computer (Sony) and a morphometry program OsteoMetrics (OsteoMetrics, Inc.). Total bone area, total bone surface, osteoid surface, osteoblast surface, osteoclast surface, eroded surface, osteoid area, and single- and double-labeled perimeters were obtained by manual tracing.

### Measurement of serum bone turnover markers

The serum bone formation markers bone-specific alkaline phosphatase (ALP), osteocalcin and IGF1 were measured using enzyme immunoassay kits from Quidel Corporation (San Diego, CA). Based on data provided by manufacture, intra- and inter-assay coefficients and sensitivity of the assay are 5.3%, 7.3% and 0.78 U/L for ALP, 5.0%, 5.5% and 50.0 ng/mL for osteocalcin, 4.3%, 8.3% and 0.026 ng/ml for IGF1, respectively. The serum bone resorption marker RatLaps ELISA kit was purchased from Nordic Bioscience Diagnostics A/S (Herlev, Denmark). According to the manufacturer's recommendation, 50 µl of serum from each sample was used and the absorbance at 450 nm with subtraction at 650 nm was measured. Intra- and inter-assay coefficients and sensitivity of the assay are 5.6%, 10.5% and 2.0 ng/mL.

### Microarray and Real-Time Reverse Transcription-Polymerase Chain Reaction

Right tibial bone total RNA and *in vitro* cultured cell RNA were extracted using TRI Reagent (MRC Inc., Cincinnati, OH) according to the manufacturer's recommendations followed by DNase digestion and column cleanup using QIAGEN mini columns. Three microarrays (GeneChip Rat 230 2.0; Affymetrix, Santa Clara, CA) were used for each group either samples from *in vivo* or *in vitro*. Pools of equal amounts of RNA from two to three rats were used for analyses per microarray, representing at least eight rats per group over the three microarrays. cRNA synthesis, labeling, hybridization, and scanning were carried out using the manufacturer's instructions and described previously in our lab [35]. Microarray data analyses were carried out using GeneSpring version 7.3X software (Agilent Technologies, Santa Clara, CA) [35]. The CEL files containing probe level intensities were processed using the robust multiarray analysis algorithm for background adjustment, normalization, and log2 transformation of perfect match values [35]. Subsequently, the data were subjected to normalization by setting measurements less than 0.01 to 0.01 and by per-chip and per-gene normalization using GeneSpring. The normalized data were used to generate a list of differentially expressed genes among different diets with and without BB. Reverse transcription was carried out using an iScript cDNA synthesis kit from Bio-Rad (Hercules, CA). Real-time RT-PCR was carried out using SYBR Green and an ABI 7000 sequence detection system (Applied Biosystems, Foster City, CA). Primers for all genes used in this report were designed using Primer Express software 2.0.0 (Applied Biosystems) and listed in a Table S1. All data is MIAME compliant and that the raw data has been deposited in a MIAME compliant database (E.g. ArrayExpress, GEO), as detailed on the MGED Society website http://www.mged.org/Workgroups/MIAME/miame.html with GSE30081.

### In vitro cell cultures and osteoblast differentiation assay

Neonatal rat calvarial osteoblastic cells were isolated from control rat 4 days old pups by sequential collagenase digestion using a method described previously [36]. Rat fetal calvarial osteoblastic cells and the mouse origin bone marrow mesenchymal stromal cell line ST2 were cultured in α-MEM supplemented with 10% FBS. Cells were seeded in six-well cell culture plates at a density of 1.5×10^6^ cells per well. Cell cultures were maintained in the presence of minimal essential medium (Invitrogen, Calsbad, CA) with 15% fetal bovine serum (FBS) (Hyclone Laboratories, Logan, UT) and 1 mM ascorbyl-2-phosphate (Sigma-Aldrich, St. Louis, MO), 4 Mm L-glutamine, and 100 U/ml each of penicillin and streptomycin (Sigma-Aldrich) as osteogenic medium. Cell cultures were stopped at indicated time points and were fixed and stained with alkaline phosphatase (ALP) and RNA and protein assays.

### Senescence-associated beta-galactosidase activity and staining

Senescence-associated beta-galactosidase activity assay was performed by beta-galactosidase enzyme assay kit (Promega) measured the absorbance at 420 nm according to manufacture's instruction. Bone section and cell beta-galactosidase staining were also performed according to a method published previously [36] Senescent cells were identified as blue-stained cells by standard light microscopy.

### Transient transfection and shRNA

ShRNA myosin (sc-149741-SH) was purchased from Santa Cruz (Santa Cruz BioTechnology, Inc, www.scbt.com), and transfection of shRNA myosin into ST2 cells were carried out using Amaxa cell line nucleofector kit (Amaxa Biosystems, www.lonza.com).

### Western blotting and immunoprecipitation

Right tibial bone tissue proteins for Western immunoblot analysis were extracted using cell lysis buffer as described previously [36]. Western blot and immunoprecipitation analyses were performed using standard protocols. Primary and secondary antibodies for myosin, beta-actin, Runx2, were purchased from Santa Cruz Biotechnology and Cell Signaling. Blots were developed using chemiluminescence (PIERCE Biotechnology) according to the manufacture's recommendations. Quantification of the intensity of the bands in the autoradiograms was performed using a VersaDoc™ imaging system (Bio-Rad).

### Data and statistical analyses

Data were expressed as means ± SEM. One way analysis of variance (ANOVA) was utilized followed by student Newman-Keuls post hoc analyses for multiple pair-wise comparisons between treatment groups. Values were considered statistically significant at p<0.05.

## Supporting Information

Figure S1 **Experimental design.** (A), Diagramed experimental design, including age, diet duration, diet switch and ovariectomy surgery time. (B). Body weight information. Experimental animals were weighed every other day.
(TIF)

Figure S2 **Triple staining of immune-staining for myosin and Runx2 and SABG activity staining in long bone sections from four different diet groups 3 weeks after OVX.** Pictures are showing typical bone surface area from sagittal section under 10 x magnifications, Green stains for myosin, red stains for Runx2 and blue stains for SABG and DAPI staining for nucleus. White arrows indicate a osteoblastic cell on bone surface. OVX, Ovariectomy; Sham, Sham operated. LTBB OVX, long term blueberry supplemented diet throughout experiment and ovariectomy. STBB OVX, short term blueberry diet for 14 days from weaning postnatal date 20 to PND 34, then switch to control diet and ovariectomy.
(TIF)

Figure S3 **Real-time PCR analysis for Myh2, 4, 7 (myosin 2, 4, 7) and ALP mRNA expression in isolated control neonatal rat calvarial cells after cells were treated with control vehicle, 2% diet animal serum from casein control (Cas) and blueberry (BB) for 24 h and 48 h.** Data are expressed as mean ± SEM (triplicates). Means with different letters differ significantly from each other at p<0.05, a<b<c.
(TIF)

Figure S4 **Original housekeeping gene normalized microarray data.** 1.5 fold up- or down-regulated genes are presented.
(TIF)

Figure S5 **Serum from BB diet animals prevents OVX-induced osteoblastic cell senescence.** (A), After ST2 cells treated one time with 2% of PBS (control), serum from three weeks of sham operated (Sham), OVX and long term BB plus OVX (BB-OVX) rats for 3d, cell cultures were switched to a regular medium until to last passage 9 (p9). Total cell numbers were counted at each passage time. (B), Proteins were isolated from passage 9 cells, and senescence associated beta-galactosidase activity was measured. (C), RNA were isolated from passage 9 cells, and mRNA expressions of ALP, Runx2 and myosin4, 7 (Myh4,7) were carried out by real-time PCR.
(TIF)

Figure S6 ***In vitro*** **cell culture model.** (A), *in vitro* cell culture experimental design. (B), After calvarial cells treated one time with 2% of PBS (control), serum from three weeks of sham operated (Sham), OVX and long term BB plus OVX (BB-OVX) rats for 3d, cell cultures were switched to a regular medium until to last passage 9 (p9). Total cell numbers were counted at each passage time.
(TIF)

Table S1 **Real-time PCR primer sequences.**
(DOC)
