# Supplementary figures and images for: Feeding Blueberry Diets in Early Life Prevent Senescence of Osteoblasts and Bone Loss in Ovariectomized Adult Female Rats
Source: PLoS One. 2011 Sep 2;6(9):e24486. doi: 10.1371/journal.pone.0024486 (PMC3166322; doi:10.1371/journal.pone.0024486)

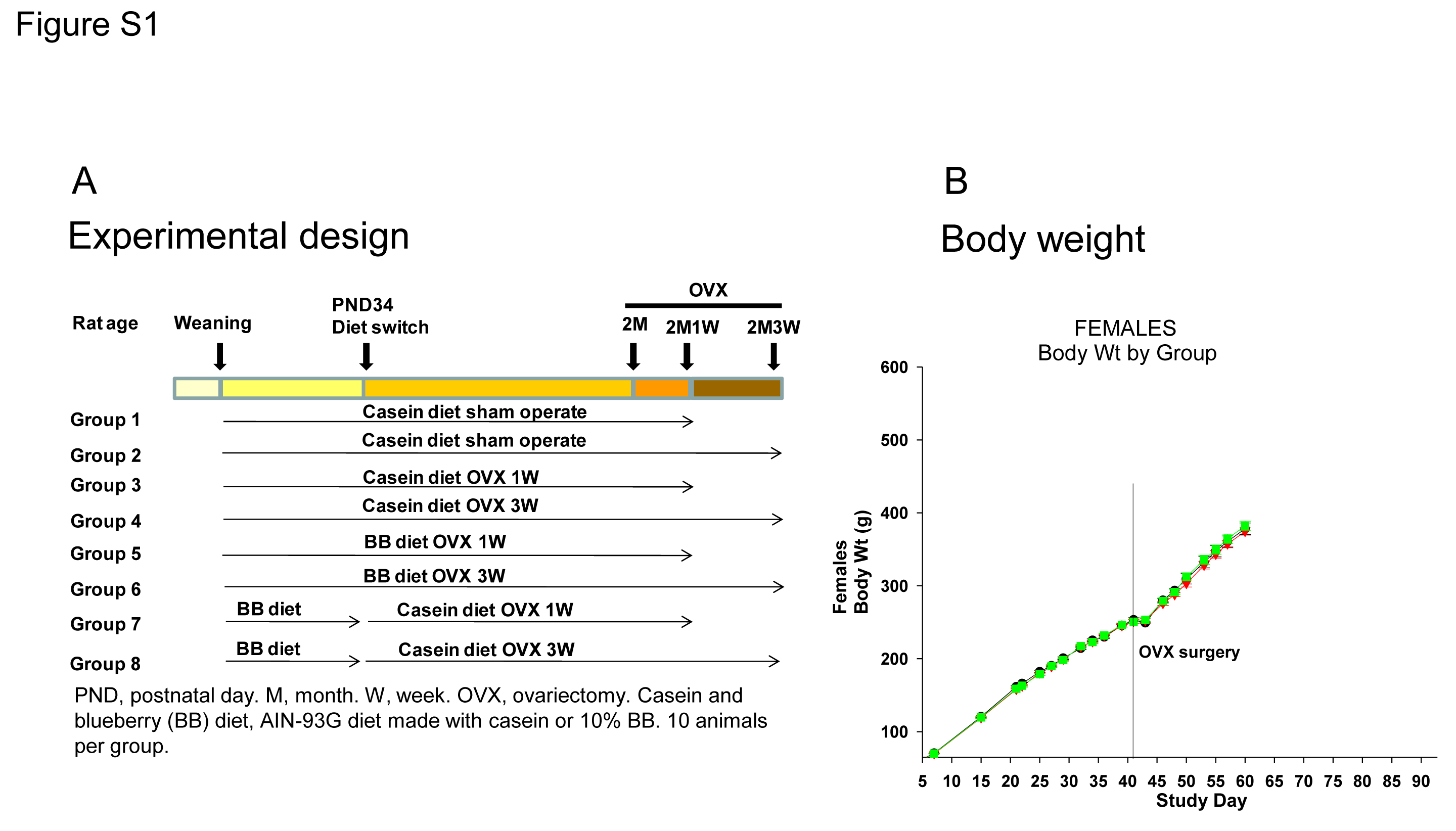

Supplement: Figure S1 — Experimental design. (A), Diagramed experimental design, including age, diet duration, diet switch and ovariectomy surgery time. (B). Body weight information. Experimental animals were weighed every other day. (TIF) [file pone.0024486.s001.tif]

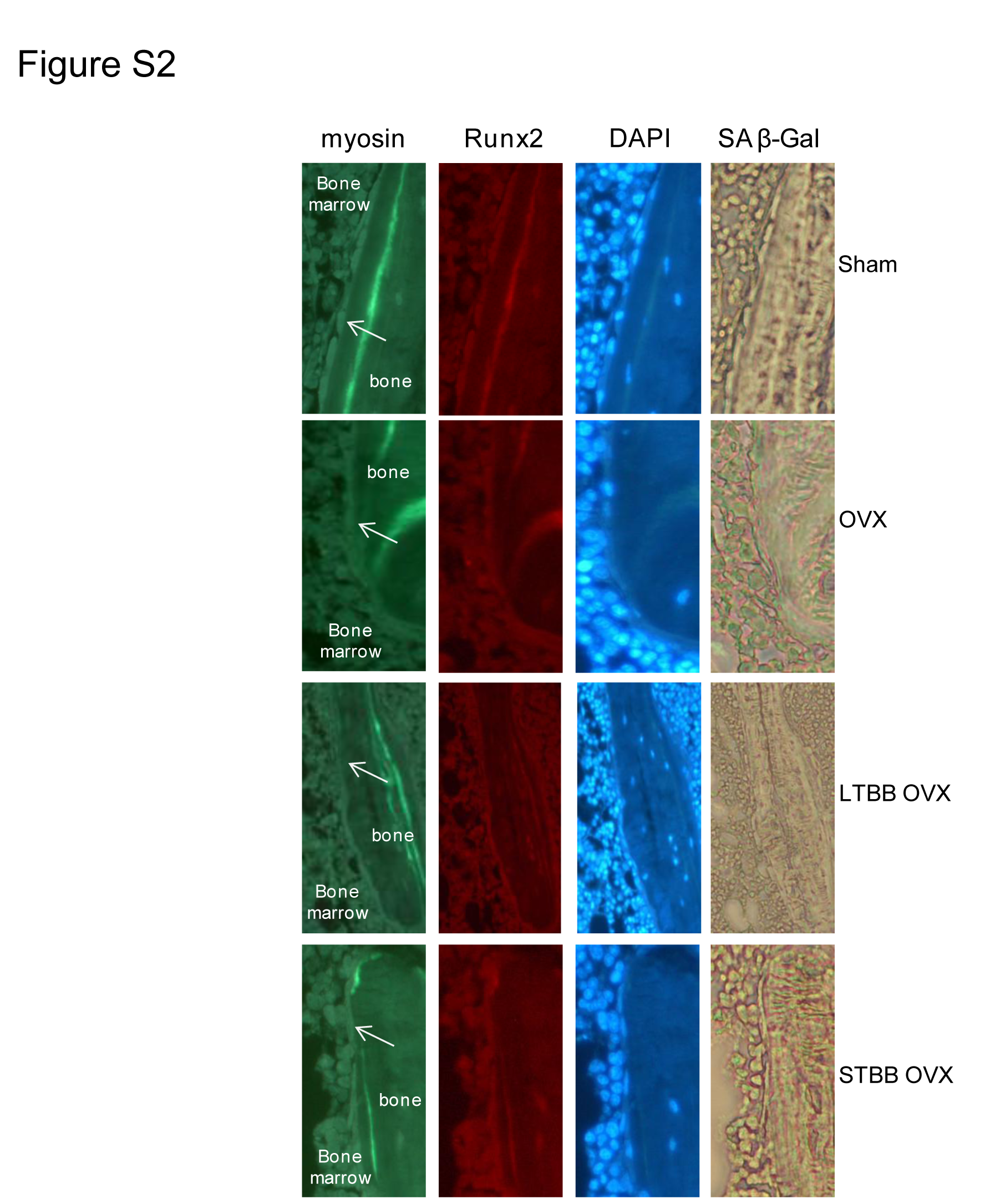

Supplement: Figure S2 — Triple staining of immune-staining for myosin and Runx2 and SABG activity staining in long bone sections from four different diet groups 3 weeks after OVX. Pictures are showing typical bone surface area from sagittal section under 10 x magnifications, Green stains for myosin, red stains for Runx2 and blue stains for SABG and DAPI staining for nucleus. White arrows indicate a osteoblastic cell on bone surface. OVX, Ovariectomy; Sham, Sham operated. LTBB OVX, long term blueberry supplemented diet throughout experiment and ovariectomy. STBB OVX, short term blueberry diet for 14 days from weaning postnatal date 20 to PND 34, then switch to control diet and ovariectomy. (TIF) [file pone.0024486.s002.tif]

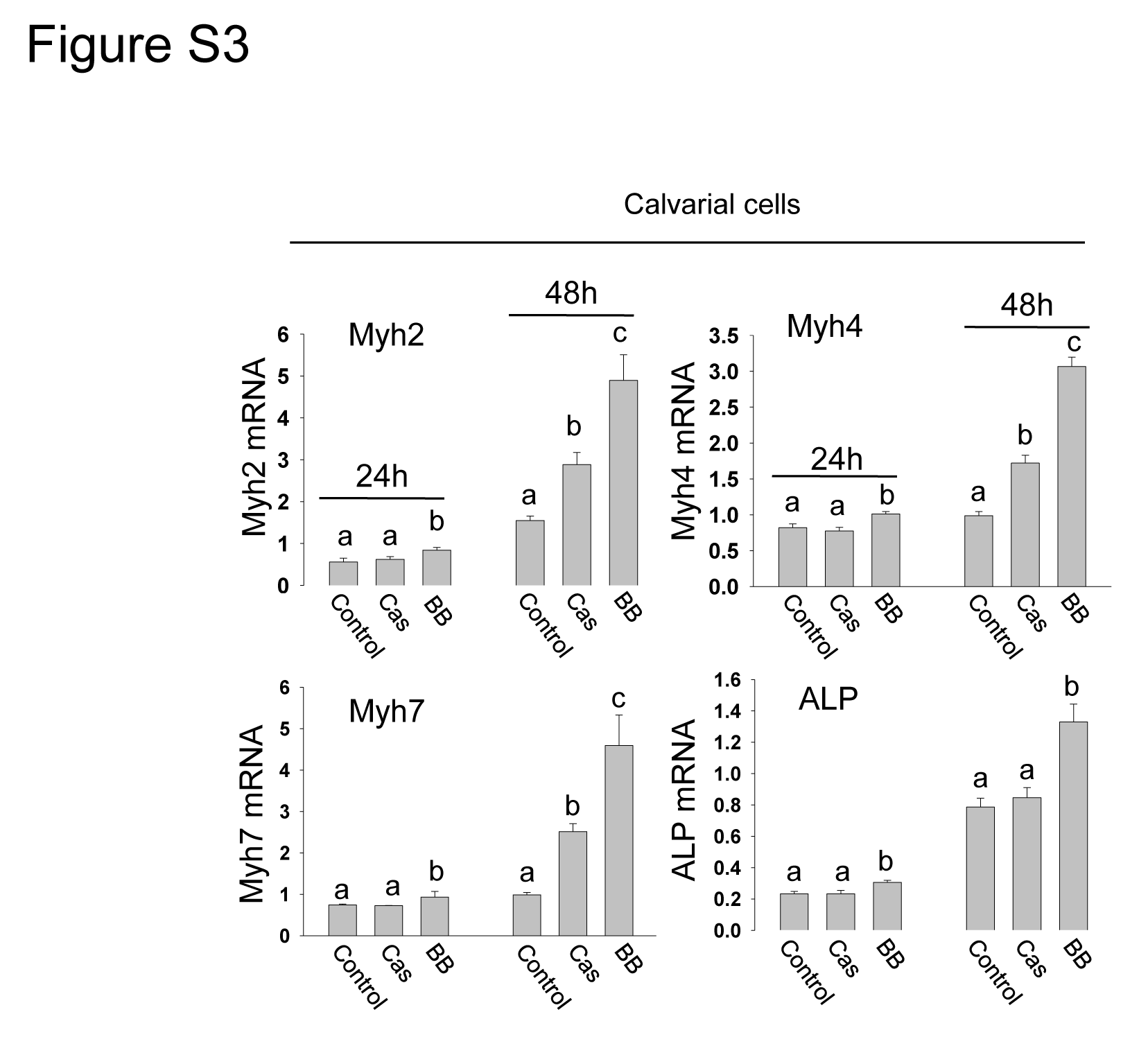

Supplement: Figure S3 — Real-time PCR analysis for Myh2, 4, 7 (myosin 2, 4, 7) and ALP mRNA expression in isolated control neonatal rat calvarial cells after cells were treated with control vehicle, 2% diet animal serum from casein control (Cas) and blueberry (BB) for 24 h and 48 h. Data are expressed as mean ± SEM (triplicates). Means with different letters differ significantly from each other at p<0.05, a<b<c. (TIF) [file pone.0024486.s003.tif]

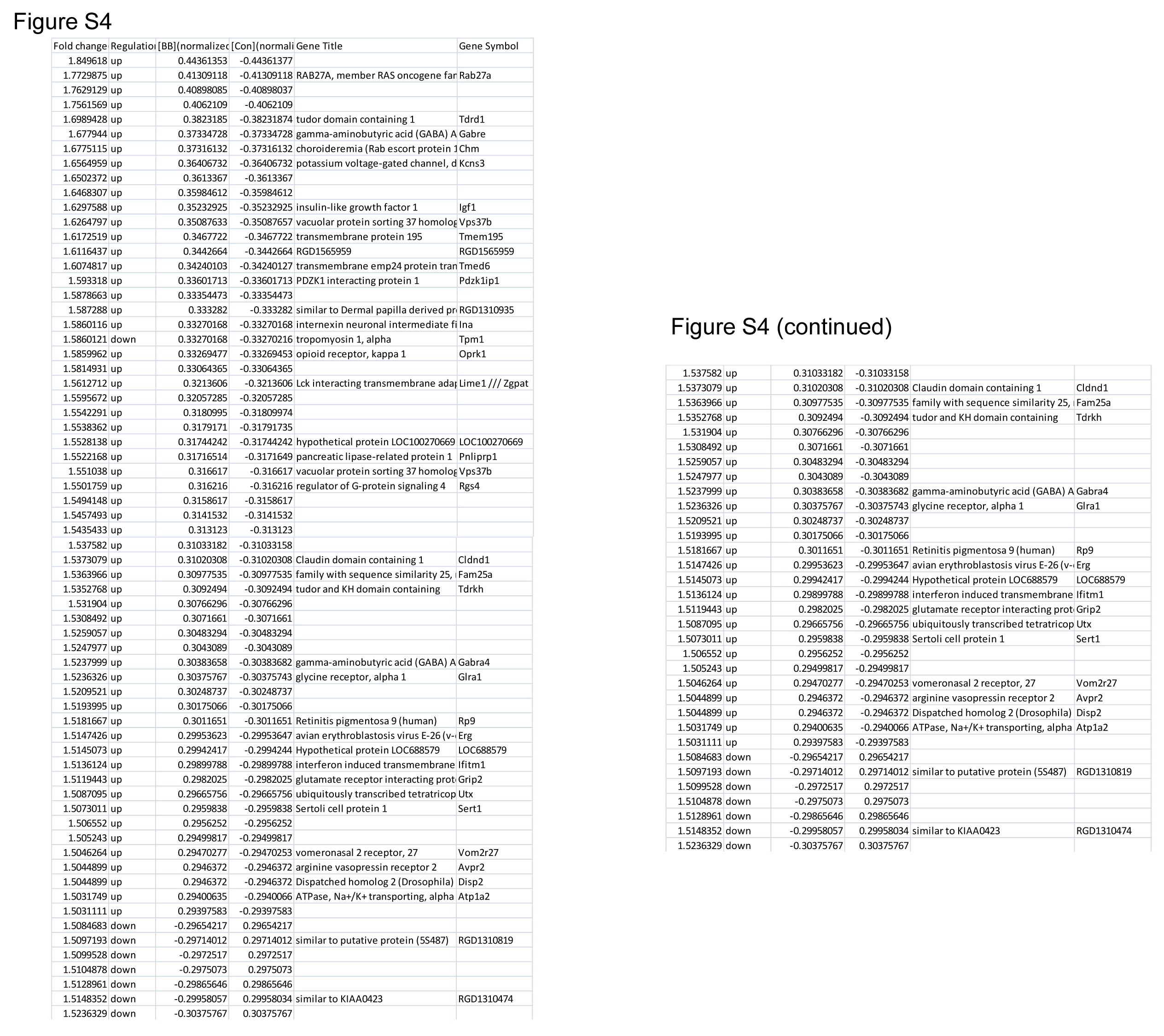

Supplement: Figure S4 — Original housekeeping gene normalized microarray data. 1.5 fold up- or down-regulated genes are presented. (TIF) [file pone.0024486.s004.tif]

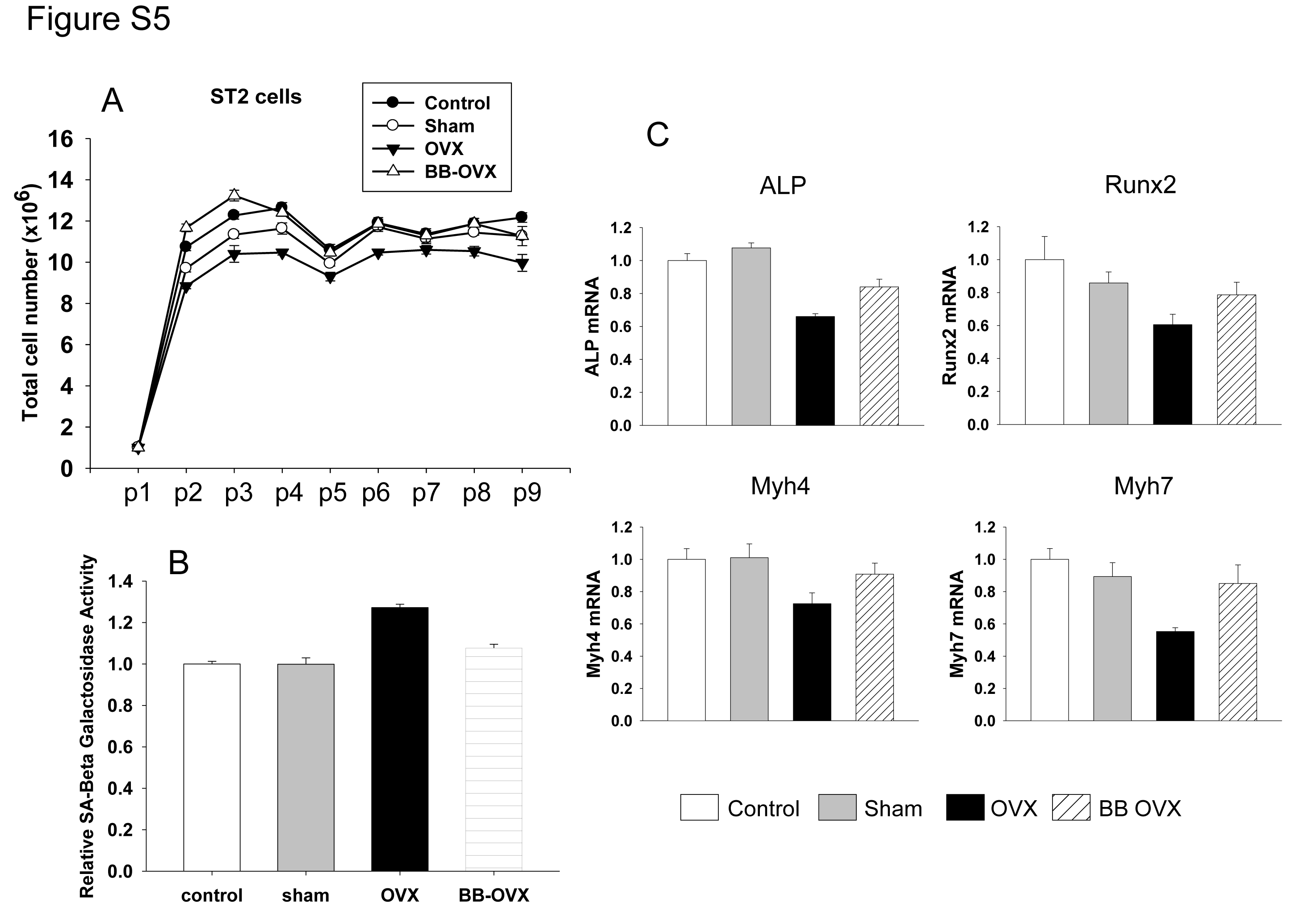

Supplement: Figure S5 — Serum from BB diet animals prevents OVX-induced osteoblastic cell senescence. (A), After ST2 cells treated one time with 2% of PBS (control), serum from three weeks of sham operated (Sham), OVX and long term BB plus OVX (BB-OVX) rats for 3d, cell cultures were switched to a regular medium until to last passage 9 (p9). Total cell numbers were counted at each passage time. (B), Proteins were isolated from passage 9 cells, and senescence associated beta-galactosidase activity was measured. (C), RNA were isolated from passage 9 cells, and mRNA expressions of ALP, Runx2 and myosin4, 7 (Myh4,7) were carried out by real-time PCR. (TIF) [file pone.0024486.s005.tif]

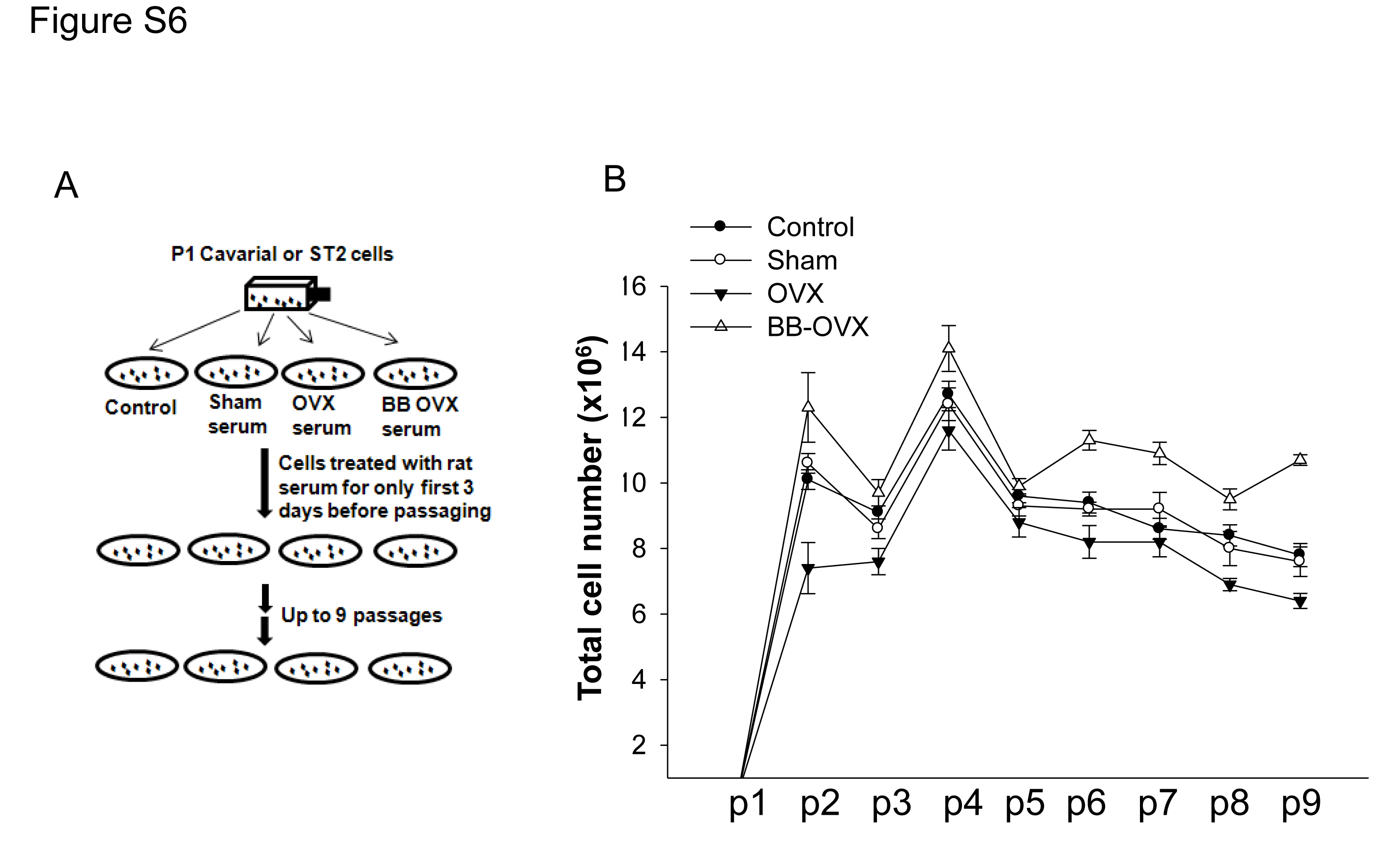

Supplement: Figure S6 — In vitro cell culture model. (A), in vitro cell culture experimental design. (B), After calvarial cells treated one time with 2% of PBS (control), serum from three weeks of sham operated (Sham), OVX and long term BB plus OVX (BB-OVX) rats for 3d, cell cultures were switched to a regular medium until to last passage 9 (p9). Total cell numbers were counted at each passage time. (TIF) [file pone.0024486.s006.tif]
