# Supplementary material for: Feeding Blueberry Diets in Early Life Prevent Senescence of Osteoblasts and Bone Loss in Ovariectomized Adult Female Rats
Source: PLoS One. 2011 Sep 2;6(9):e24486. doi: 10.1371/journal.pone.0024486 (PMC3166322; doi:10.1371/journal.pone.0024486)
Supplement: Table S1 — Real-time PCR primer sequences. (DOC) [file pone.0024486.s007.doc]

Table S1. Real-time PCR primer sequences

| Gene | | Forward primer | Reverse primer |
| --- | --- | --- | --- |
| Rat |  |  |  |
|  | ALP | TGAATCGGAACAACCTGACTGA | TTCCACTAGCAAGAAGAAGCCTTT |
|  | Runx2 | CCGTGGCCTTCAAGGTTGTA | ATTTCGTAGCTCGGCAGAGTAGTT |
|  | osteocalcin | AAGCCCAGCGACTCTGAGTCT | GCTCCAAGTCCATTGTTGAGGTA |
|  | myh1 | CATGAACCCTCCCAAGTACGA | AGGCTGCGTAACGCTCTTTG |
|  | myh2 | CGAATCGAGGCCCAGAATAG | GCGTCTTTGCTCTGAATGGTT |
|  | myh3 | GCTCAGAACCAGCCCTTTGA | ACTTTCCCATCCTGGCTACTCTT |
|  | myh4 | ATCAGTGTTTGTGGTGGATGCTAA | CAGTAGCTCCACCTTCGGTCTT |
|  | myh6 | ACCACCTGGGCAAGTCCAA | GTAGTCCACGGTGCCAGCAT |
|  | myh7 | GAGTATGTCACCAAAGGGCAGAAT | GCGTGTCACCATCCAGTTGA |
|  | Cyclophilin | AGCATACAGGTCGCATCT T | GCCATCGAGCCACTCAGTCT |
|  |  |  |  |
| Mouse |  |  |  |
|  | ALP | CCAATGTAGCCAAGAATGTCATCA | GCCCGGTGTGGTGTAGCT |
|  | Runx2 | CGGTCTCCTTCCAGGATGGT | GCTTCCGTCAGCGTCAACA |
|  | myh1 | CCTAGCCAAAGCCGTCTATGA | CCAAGACCCCGATGAAGTACTG |
|  | myh2 | CTGTATGAGGAGCATCTTGGAAA | GTGCCCGCATAGTGGATGA |
|  | myh3 | TCCGACAACGCCTACCAGTT | TGACCCGCTTGGTGTTCAC |
|  | myh4 | TCTCCATGAACCCTCCCAAGT | TGCATAACGCTCTTTGAGGTTGT |
|  | myh6 | GCTGACAGATCGGGAGAATCAG | TGCAATGCTGGCAAAGTACTG |
|  | myh7 | AGATGTTCAACTGGATGGTGACA | GAGATCTCAAAGCCGGCAAT |
|  | Cyclophilin | AAG GTG GAG AGC ACC AAG ACA | GCA ATG GCG AAG GGT TTC T |

Primers for all genes used in this report were designed using Primer Express software 2.0.0 (Applied Biosystems).
